# Research progress in treatment of rheumatoid arthritis with Sinomenine and related formulations based on different administration routes

**DOI:** 10.3389/fphar.2025.1613679

**Published:** 2025-08-06

**Authors:** Wenya Wang, Zihui Wang, Aixia Ling, Chunyan Zhang, Mei Lv, Lufen Huang, Yanlian Niu

**Affiliations:** School of Pharmacy, Jining Medical University, Rizhao, China

**Keywords:** Sinomenine, mechanism of action, related formulations, drug delivery system, rheumatoid arthritis

## Abstract

Sinomenine (SIN), a multi-target alkaloid extracted from *Sinomenium acutum*, demonstrates significant immunomodulatory, anti-inflammatory, and osteoprotective properties in the treatment of rheumatoid arthritis (RA). It achieves these effects by modulating immune cells, such as macrophages and T cells, suppressing pro-inflammatory cytokines like tumor necrosis factor-α (TNF-α) and interleukin-6 (IL-6), and inhibiting synovial hyperplasia and bone erosion. Recent advancements in drug delivery systems (DDSs), including oral sustained-release formulations, transdermal microneedles, lipid-based carriers (such as transfersomes and ethosomes), and intra-articular thermosensitive hydrogels, have markedly improved its bioavailability, targeting precision, and therapeutic longevity. For example, reactive oxygen species-responsive microneedles and biomimetic nanocarriers facilitate spatiotemporal-controlled drug release, while hybrid exosome-liposome systems enhance synovial retention and minimize systemic toxicity. Although preclinical results are promising, challenges like incomplete clinical validation, limited exploration of combination therapies, and inadequate adaptation to RA’s dynamic microenvironments persist. Future research should focus on developing intelligent DDSs with multi-stimuli responsiveness, leveraging omics for mechanistic insights, and creating patient-specific delivery strategies to enhance clinical application. This review highlights SIN’s transformative potential in RA management and calls for interdisciplinary collaboration to improve its translational success.

## Graphical Abstract

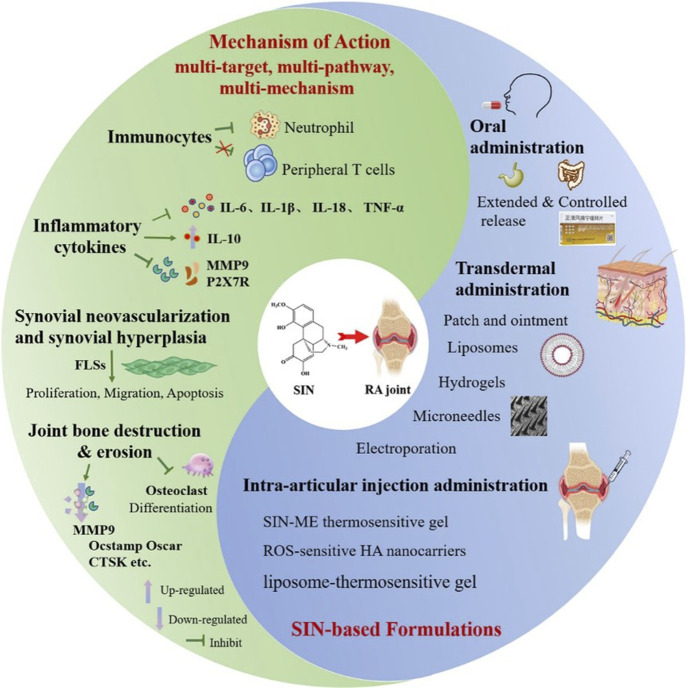

## Highlights

• This manuscript provides a detailed and systematic review of the multi-target pharmacological action of SIN against RA pathogenesis.
• This article researches efficient DDSs based on transdermal, oral, and intra-articular injection of administration. The innovative formulations discussed can achieve spatiotemporal-controlled release, enhancing bioavailability, and facilitating precise targeting of lesion sites.
• The challenges in translating SIN and SIN-based DDSs to clinical use have been innovative prospected.

## 1 Introduction

Rheumatoid arthritis (RA) is a chronic systemic autoimmune disease in which synovitis is the basic pathological change, and the main sites of development are the proximal interphalangeal joints of both hands, the metacarpophalangeal joints, the wrists, the knees, and the feet, and it is often accompanied by joint swelling, induration, and rigidity during the active period (Zhang C. et al., 2022; Hurysz and Bottini, 2022). According to statistics, the global prevalence of RA is 0.5%∼1%, and the prevalence of women is about 2∼3 times higher than that of men. The prevalence increases significantly with age, and the highest prevalence is found in elderly people over 65 or 70 years old (Boutet et al., 2021). The therapeutic approach for RA emphasizes early diagnosis and the management of disease progression. Common pharmacological interventions encompass nonsteroidal anti-inflammatory drugs (NSAIDs), disease-modifying antirheumatic drugs (DMARDs), glucocorticoids, and biological agents (Li et al., 2023). Nonetheless, prolonged administration of these medications is frequently associated with a spectrum of adverse effects. For instance, while glucocorticoids and NSAIDs are efficacious in alleviating pain and inflammation, the side effects long-term use are significant, such as a high incidence of heart disease and stroke (Ozen et al., 2023). Methotrexate (MTX), the drug of choice, is often accompanied by adverse effects such as nausea, vomiting, oral ulcers, and hepatotoxicity (Chen et al., 2022b). Similarly, leflunomide (LEF) is associated with side effects including diarrhea, nausea, headache, rash, pruritus, alopecia, hypertension, chest pain, palpitations, infections, and hepatic dysfunction (Behrens et al., 2021; Chen et al., 2022b). Furthermore, biomacromolecular anti-RA agents with non-negligible increasing cost burden, such as tumor necrosis factor α (TNF-α) inhibitors, are frequently linked to infections and allergic reactions (Chen et al., 2022b; Li et al., 2023).

**FIGURE 1:**
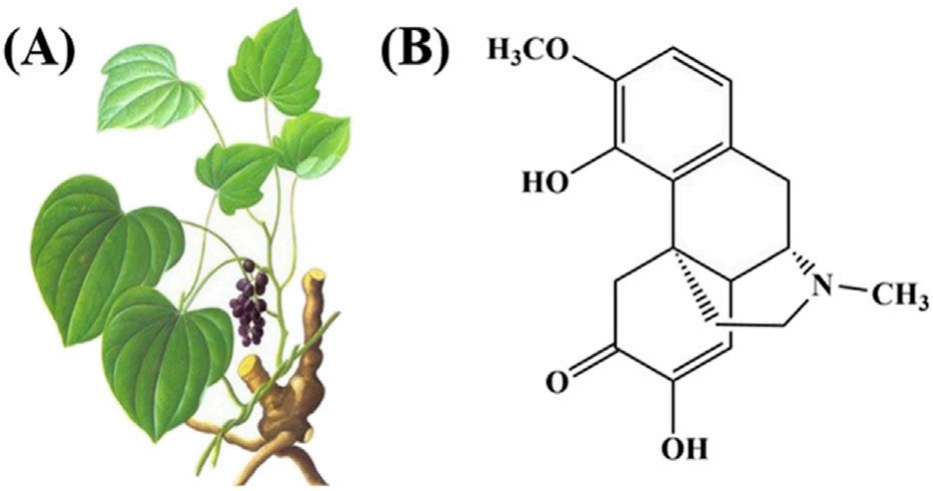
**(A)** The picture of Sinomenium acutum (Thunb.) Rehd.et Wils. and **(B)** the structural formula of SIN.

Traditional Chinese Medicine (TCM) treatment of RA is another effective choice for clinical application (Liu et al., 2022), “Compendium of Materia Medica” recorded that Qingfengteng can be “steeped in medicinal wine for therapeutic use in treating” to treat “rheumatic wandering arthritis (*fengshi liuzhu*), severe joint swelling (*lijie hexi*), numbness and itching (*mabi saoyang*), traumatic injuries with ulcerative swelling (*sunshang chuangzhong*)” (Zhang E. et al., 2022). Sinomenine (SIN) is an alkaloid compound isolated from the roots and stems of the TCM Qingfengteng (Figure 1), and numerous studies have shown that SIN has analgesic, anti-inflammatory, immunomodulatory, and inhibition of graft rejection and other pharmacological effects (Liu et al., 2022; Li et al., 2025). Furthermore, clinical randomized controlled trials have demonstrated that SIN exhibits efficacy comparable to MTX in the treatment of patients with RA, while presenting with fewer adverse effects (Shi et al., 2022). In detail, in the SIN-treated group, 52.63% of patients achieved the American College of Rheumatology (ACR) 50 after 24-weeks of treatment, which was comparable to the results in the MTX-treated and SIN+MTX-treated groups. Hepatic and gastrointestinal disorders were the main adverse events; however, the ratio of patients suffering from hepatic disorder in the SIN group (1/38) was much lower than that in the MTX (10/39) and SIN+MTX (8/36) groups. Additionally, patients receiving a combination of SIN and MTX experienced reduced gastrointestinal side effects and liver toxicity compared to those treated with MTX+LEF (Huang et al., 2019). These research results suggest that SIN possesses ideal efficacy and safety profiles for anti-RA therapy.

The primary clinical forms of SIN are Sinomenine hydrochloride (SIN-HCl) tablets, enteric-coated tablets, and injections. SIN is prone to unstable, decomposing easily under alkali, light, and heat, with a short half-life and low bioavailability. These issues necessitate high doses for long-term treatment and can lead to side effects like histamine release, skin rashes, and gastrointestinal reactions (Wang et al., 2023).

This article systematically reviews the mechanism of SIN in anti-RA, the research progress of SIN and its preparations over the past 5 years, and the development of new SIN delivery systems based on various administration routes, aiming to guide related research and n novel drug delivery systems (DDSs) development.

## 2 Mechanism of action

### 2.1 Effects on immunocytes

Immunocytes are cells involved in or associated with the body’s immune response, including dendritic cells, macrophages, various granulocytes and so on. A growing number of studies have shown that various immune cells, accompanied with the relative inflammatory factors are involved in the pathogenesis of chronic pain, and SIN has a powerful inhibitory effect on chronic pain (Lai et al., 2022; Yu et al., 2024). Using Western blotting and immunofluorescence assays, Jiang et al. found that SIN had an inhibitory effect on the migration of neutrophils. And upon activation of lipopolysaccharide (LPS) *in vitro*, SIN suppressed the phosphorylation of P65, extracellular signal-regulated kinase (ERK) and P38 of neutrophil. Meanwhile, SIN inhibited neutrophil extracellular traps (NETs) formation induced by phorbol 12-myristate 13-acetate (PMA), which were demonstrated by the decreased expression of neutrophil elastase, protein arginine deiminase type 4 (PAD4) and ctrullinated histone H3 (CitH3). All of the above suggest that SIN has good efficacy in treating adjuvant-induced arthritis (AIA) via regulating neutrophil activities (Jiang H. et al., 2023). However, by comparing the effects of 10 ng/mL methylprednisolone and 0.3 μM ∼ 30 μM SIN on mitogen-activated human peripheral blood mononuclear cells (PBMCs), Sugiyama found that even with 30 μM SIN, the ability to secrete Th1/Th2/Th17 cytokines was not significantly affected, suggesting that SIN’s anti-RA mechanism does not appear to be related to the inhibitory effect of peripheral T cells (Xu et al., 2021).

### 2.2 Inhibition of inflammatory cytokines

Inflammatory cytokines refer to a class of cytokines that can induce T cell activation, proliferation and differentiation and other related response responses. The anti-inflammatory mechanism of SIN has been systematically elucidated in several studies, and its core mechanism involves multi-targeted modulation of the inflammatory cytokine network and monocyte/macrophage subpopulations. In a collagen-induced arthritis (CIA) model and RA patients, SIN significantly modulated monocyte/macrophage-mediated inflammatory responses by inhibiting the secretion of pro-inflammatory factors (e.g., interleukin-6 (IL-6), TNF-α, and interleukin-1β (IL-1β)) and upregulating the expression of anti-inflammatory factors (e.g., interleukin-10 (IL-10)), thereby delaying the progression of RA (Zeng and Tong, 2020; Li R. Z. et al., 2023; Li J. M. et al., 2023). Further *in vitro* studies demonstrated that SIN inhibited LPS-induced overexpression of matrix metalloproteinase 9 (MMP9) protein, TNF-α, and IL-6 in RAW264.7 macrophages, suggesting that it attenuates joint damage by inhibiting matrix metalloproteinases and pro-inflammatory factor release (Luo et al., 2024). In addition, molecular mechanism studies revealed that SIN competitively bind to guanylate-binding protein 5 (GBP5), downregulate the expression of P2X7R protein, and then inhibit the activation of the NLRP3 inflammatory vesicle pathway, which ultimately reduces the production of key inflammatory mediators such as IL-1β and interleukin-18 (IL-18) (Li et al., 2023).

Numerous researchers have systematically analyzed the anti-inflammatory targets of SIN from cellular, animal model and molecular levels, especially emphasizing its regulation of monocyte/macrophage subpopulations and the balancing effect of inflammatory factor networks. Available evidence suggests that the anti-inflammatory mechanism of SIN is multi-targeted, covering the inhibition of classical pro-inflammatory factors, enhancement of anti-inflammatory factors, regulation of matrix-degrading enzymes, and intervention of inflammatory vesicle pathways. However, most of the current studies focus on known inflammatory pathways, and whether SIN act through other signaling nodes for anti-RA still needs to be explored in depth.

### 2.3 Inhibition of synovial neovascularization and synovial hyperplasia

Neovascularization and synovial hyperplasia accompany RA throughout the course of the disease, and SIN exhibit multidimensional regulatory effects in inhibiting synovial hyperplasia and neovascularization in RA, with the mechanisms centering around the functional intervention of fibroblast like synoviocytes (FLSs). SIN could selectively inhibit the abnormal proliferation of RA-FLS by inhibiting the phosphorylation of AMPK, inducing the phosphorylation of Ser349 and Thr269/Ser272 sites of p62, and activating the Keap1-Nrf2 antioxidant signaling pathway in FLS, while having no effect on normal macrophages (Liao et al., 2021; Li et al., 2023), and further studies revealed that SIN may inhibit LPS-induced pro-inflammatory cytokine release and abnormal invasive migration of FLSs by CRMP2 T514 phosphorylation and its nuclear translocation of FLSs, suggesting both anti-inflammatory and analgesic potentials (Yu et al., 2024). In addition, SIN significantly inhibited NETs-mediated RA-FLS migration and blocked the pro-synovial proliferative effects of PDGF)/PDGFR signaling pathway by down-regulating the expression of PDGFRβ and MMPs (MMP1/3/9) (Zhang et al., 2024). At the level of molecular regulation, SIN promotes RA-FLS apoptosis by up-regulating microRNA-23b-3p and fibroblast growth factor 9 (FGF9) and balances the expression of pro-inflammatory factors (TNF-α, IL-1β) and anti-inflammatory factors (arginase 1 (Arg-1), IL-10) with the help of nuclear factor kappa-B (NF-κB) pathway (Yi et al., 2021; Shang et al., 2023). Network pharmacology combined with experimental validation further revealed that SIN can block the pathological activation of RA-FLS by inhibiting the activity of PI3K/Akt signaling pathway (Liu Q. et al., 2024).

The reported studies systematically elucidated the multi-targeted regulatory properties of SIN in RA synovial pathology, and its effects cover multiple key aspects of FLS proliferation, migration, apoptosis, and remodeling of inflammatory signaling networks. In particular, SIN synergistically inhibits synovial proliferation and neovascularization by targeting GBP5/P2X7R-NLRP3 (Li et al., 2023), AMPK/Keap1-Nrf2 (Liao et al., 2021; Li et al., 2023), CRMP2 (Yu et al., 2024), PDGF/PDGFR (Zhang et al., 2024), NF-κB (Yi et al., 2021; Shang et al., 2023) and PI3K/Akt pathways (Liu Q. et al., 2024), which provides a new perspective on RA pathology. Sun et al. (Zhang et al., 2024) focused on the interactions between NETs and FLS, which broadened the understanding of the complexity of the RA microenvironment. However, the mechanism of crosstalk between different signaling pathways (e.g., cross-regulation of Nrf2 and NF-κB) has not been clarified by SIN; furthermore, the targets of SIN’s direct modulation of synovial vascular neogenesis (e.g., the vascular endothelial growth factor (VEGF) pathway) still need to be explored in depth.

### 2.4 Inhibition of joint bone destruction and erosion

Bone destruction is one of the major causes of joint deformity, stiffness and dysfunction in RA patients. SIN and its derivatives exhibit significant pharmacological potential in inhibiting bone destruction in RA, and their mechanism of action involves the synergistic intervention of osteoclast differentiation inhibition and synovial pathology modulation. Moreover, the release of key pro-inflammatory cytokines secreted by activated macrophage induces rapid proliferation and division of FLSs, and causes cartilage matrix disintegration and bone destruction (Alivernini et al., 2020; Nygaard and Firestein, 2020). Thus, a biomimetic nanocomplex based on Prussian blue nanoparticles (HA@M@PB@SIN NPs) was developed by Cai’s team, which was showed excellent biocompatibility and favorable safety profile. Notably, *in vitro* studies demonstrated that HA@M@PB@SIN NPs significantly suppressed joint inflammation and protected against bone destruction of AIA rats by inhibiting abnormal proliferation of FLSs via scavenging reactive oxygen species (ROS) and inhibiting secretion of proinflammatory cytokines (Lin et al., 2022). Pan successfully obtained a SIN derivative (SINX) by modifying SIN A ring 1 position through a series of reactions. SINX (IC_50_ = 32.94 μM) targets and inhibits the entire process of osteoclast differentiation. *In vitro* (at a safe concentration<12.5 μM), 2.5 μM SINX significantly TRAP-positive cells (almost disappearing), disrupts the formation of F-actin rings, and completely inhibits the activity of bone resorption lacunae. Additionally, SINX regulates the expression of key osteoclast genes (such as Ocstamp, Oscar, CTSK, MMP9, etc.) in a dose- and time-dependent manner, significantly RANKL-induced osteoclast differentiation and function. At a concentration of 2.5 μM, it almost completely blocks the entire differentiation process from early to terminal stages. Moreover, SINX can significantly reduce the levels of pro-inflammatory factors (TNF-α, IL-1β, IL-6) secreted by RA-FLS *in vitro*, which mediate synovial inflammation and activate osteoclasts. In the CIA mouse model, SINX (25, 50 mg/kg/d) significantly improves joint morphology, maintains trabecular bone density, reduces inflammatory infiltration and bone erosion, and reduces osteoclast activity by inhibiting the expression of genes related to bone destruction, thereby protecting bone integrity. In contrast, SIN is effective but requires a higher concentration (500 μM *in vitro*; 100 mg/kg/d *in vivo*) (Guo et al., 2025). In addition, the nanomedicine systems (HA@RFM@GP@SIN NPs) prepared by Lin’s team enhanced the anti-RA efficacy through the synergistic effect of multiple pathways: on the one hand, it regulated the metabolic reprogramming processes such as steroid hormone biosynthesis and tryptophan metabolism, on the other hand, it induced the G2-phase arrest of the RA-FLS cell cycle by inhibiting the PI3K/Akt/SGK/FoxO signaling cascade (accompanied by downregulation of cell cycle protein B1 expression), thus effectively inhibiting synovial proliferation, cartilage destruction and bone erosion in AIA and CIA models (Lin et al., 2024).

In summary, SIN mainly treats RA by regulating immunity, anti-inflammation, cell growth and apoptosis, bone metabolism and signaling pathways, etc. For example, SIN can reduce the levels of pro-inflammatory factors such as IL-6, IL-18, TNF-α, and IL-1β (Lin et al., 2022; Li et al., 2023; Shang et al., 2023; Luo et al., 2024), and increase the levels of anti-inflammatory factors such as Arg-1, and IL-10 (Shang et al., 2023), so as to achieve the purpose of reducing inflammatory response; secondly, SIN can modulating the signaling pathways such as, TLR4/NF-κB (Zeng and Tong, 2020); AMPK/Keap1-Nrf2 (Liao et al., 2021; Li et al., 2023; Hou et al., 2024), CRMP2 (Yu et al., 2024), PDGF/PDGFR (Zhang et al., 2024), PI3K/Akt pathways (Liu Q. et al., 2024), NF-κB/MAPK (Feng et al., 2025) etc., and slow down the proliferation of FLS cells, thus reducing inflammation. At the same time, studies have shown that SIN have an inhibitory effect on both T- and B-cell activation, and they can also reduce the molecular expression of the intracellular cytokines TNF-α in T-cells (Shang et al., 2023; Luo et al., 2024). Furthermore, SIN can stimulate osteoclasts to increase the secretion of osteoprotegerin, improve the ratio of osteoprotegerin to RANKL (Li et al., 2023; Guo et al., 2025), promote the differentiation and maturation of osteoblasts, inhibit bone destruction, and play an osteoprotective role. All of the above can show that the treatment of RA with SIN is characterized by multi-target, multi-pathway and multi-mechanism.

## 3 Development of SIN-based formulations for effective RA therapy

In contrast to conventional dosage forms, TCM can be innovatively reformulated into nanomedicine through the integration of advanced nanotechnology. This transformation offers several advantages, including improved bioavailability, targeted drug distribution *in vivo*, sustained and controlled release mechanisms, enhanced therapeutic efficacy, and reduced toxicity (Zheng et al., 2022). Herein, based on different routes of administration, such as oral administration, transdermal administration and intra-articular injection, the research progress of SIN-related formulations in anti-RA was reviewed detailly (Table 1).

**TABLE 1:** The table of SIN-based formulations in the treatment of RA.

Style of delivery	Design/manufacturing methods	Outstanding properties	References
Patch and ointment	combined acupoint patch therapy with patches	acupoint injection greatly improved therapeutic efficacy	Bai et al. (2022)
	3% volatile oil was used to penetration enhancers	outstanding osmotic-promoting effect	Dumitriu et al., 2023; Fan et al., 2023
Intravenous	synovial-targeting peptides conjugated with SIN	excellent stability and targeting selectivity, effective in alleviating acute inflammation	Zhang et al. (2022c)
	graphene oxide quantum dot composite nano-systems (HA@RFM@GP@SIN NPs)	Synovial membrane targeting, high drug loading capacity, promoting M1 polarization towards M2, inhibiting abnormal proliferation of FLSs	Lin et al. (2024)
	thermosensitive liposomes (SIN-TSL) with a high SIN-HCl encapsulation rate by the pH-gradient method	combined with the microwave hyperthermia technology to achieve precise controlled release of the RA lesion and hyperthermia synergistic treatment	Shen et al. (2020)
Liposomes	liposomes with [Arg][Dec] as vesicles	a higher encapsulation rate (83.5%), stability (90.4%), and transdermal absorption capacity (cumulative release of up to 1665.59 μg/cm^2^), and long-lasting sustained release performance	Wang et al. (2024)
	hybridization of milk exosomes and liposomes	reduce TNF-α, IL-1β and IL-6 levels in CIA model rats, while prolonging the short half-life of SIN	Wen et al. (2024)
	PEGylated transfersomes utilize the dermato-compatibility of phosphatidylcholine to target SIN to the joint cavity	satisfied the need for deep delivery, highlighting the penetration and accumulation	Zheng et al., 2020; Akram et al., 2021; Munir et al., 2024
Hydrogels	lipid-based liquid crystal gel with the lipophilic pro-osmotic agent cinnamaldehyde	realized the efficient co-loading of hydrophilic/lipophilic drugs	Chen et al. (2022a)
	SIN microemulsion gel (SMG) by virtue of two-site microdialysis	form a long-lasting reservoir effect in the skin (AUC skin/blood ratio of 1.73:1)	Ding et al. (2022)
	liposome-hydrogel composite loaded SIN with the synergistic effect of dual carriers	enhanced the drug stability and the target tissue accumulation capacity	Tian, 2021; Yang et al., 2022
Microneedles	near-infrared responsive SIN-HCl reservoir microneedle	a drug loading capacity of 0.5 mg/cm^-2^, and deliver the SIN to dermis layer of the skin with a puncture depth of 300 μm	Wang et al. (2022)
	ROS-responsive nanoparticles coupled with fucoidan microneedle system (FTL@SIN MNs)	satisfactory mechanical strength and stability because of the fucoidan, alleviated synovial inflammation and promoted cartilage repair	Liu et al. (2024b)
	ROS-responsive microencapsulated dissolvable microneedle (B/S-TM@MN) co-loaded with berberine and SIN	achieved synergistic release, inhibited synovitis, and neovascularization in CIA mice	Hua et al. (2025)
	two-layer PVP/PLP microneedles (SIN@PLP MNs) with biphasic releasing of SIN	achieved immediate analgesia and sustained synergistic anti-inflammation in an AIA rat model	Feng et al. (2025)
Intra-articular injection administration	liposome-thermosensitive gel conjugation	enhanced the bioavailability of SIN to 2.0-fold of the injectable solution, prolonged the time to peak by 3.0-fold, which resulted in long-lasting and slow-release with pharmacokinetic optimization	Wang et al. (2020)
	anocrystalline self-stabilized Pickering emulsion with solid liposome nanoparticles	reduced toxicity and improves bioavailability by forming a local drug reservoir	Zhang et al., 2022d; Zhang et al., 2023
	SIN-ME thermosensitive gel with *T*_sol-gel_: 35.2°C ± 1.33°C	achieved a cumulative drug release rate of 66.7% ± 6.2% at 48 h	Niu et al. (2024)
	ROS-sensitive HA nanocarriers (PAM-HA@SIN NPs) by virtue of the bone adsorption and ROS scavenging function to realize “drug in therapeutic polymer” design strategy	extended the retention time of SIN in inflamed joints to more than 20 days, and successfully remodel the oxidative/inflammatory microenvironment in RA	Shang et al. (2023)
	integrating photothermal/photodynamic properties to construct a bionic DDS (CIPG/SH)	achieved synergistic macrophage proliferation inhibition and antimicrobial therapy triggered by the near infrared	Gong et al. (2024)

### 3.1 Oral administration

Oral drugs are popular due to their easy preparation, transport, storage, and use. The State Drug Administration lists 16 solid forms of SIN-HCl, including tablets and capsules. While traditional tablets can irritate the gastrointestinal tract, advanced forms like extended-release and enteric-coated tablets reduce such effects (Chen et al., 2022b; Rivera et al., 2023). Novel oral dosage forms, such as pharmaceutical co-crystals, have improved drug bioavailability and efficacy (Rajendran et al., 2021). However, there is still necessary for oral formulations with longer-lasting sustained-release effects. Additionally, optimizing and thoroughly testing SIN tablets and injections is crucial to minimize side effects.

#### 3.1.1 Extended release formulations

Innovations in oral extended-release formulation technology have significantly optimized the pharmacokinetic properties of SIN for the treatment of RA. Research has demonstrated that the preparation parameters-specifically the drug-excipient (e.g., hydroxypropyl methylcellulose (HPMC) backbone system) ratio, pressure, and mixing time-of SIN sustained-release tablets can be effectively optimized to balance the drug release rate with the feasibility of the tablet manufacturing process, but without addressing the risk of burst release (Zeng et al., 2022).

On the other hand, microencapsulation technology, provides an ideal carrier strategy for oral delivery of SIN by protecting the drug stability and reducing gastrointestinal irritation through physical barriers, simultaneously realizing the controlled release and bioavailability enhancement (Gan et al., 2022).The dual-release micro-pellet system designed by Li’s team (conventional sustained-release and enteric sustained-release) was characterized by a high encapsulation rate (83.36%), excellent physical properties (circularity of 0.971, particle size span of 0.808) and batch-to-batch consistency (relative standard deviation = 3.26%) to achieve 12-h stable dissolution, highlighting its potential for industrial production (Wu et al., 2024). Sun et al. used cyclone fluidized bed technology to prepare extended-release microspheres, which significantly improved microsphere yield (>96%) and encapsulation rate (>90%) compared with the traditional process, and multiple batches of capsules demonstrated a 12-h smooth release behavior, which confirms the technical advantages of this technology in the large-scale production of extended-release formulations. This confirms the technical advantages of this technology in the scale production of extended-release formulations (Wu et al., 2022).

The technological breakthrough of oral sustained-release formulations provides a key solution for the long-term RA management of SIN. Existing studies have systematically solved the bottlenecks such as short half-life of SIN and high fluctuation of blood drug concentration through the optimization of backbone materials, microencapsulation process, and novel micro-pill/micro-sphere design. Among them, the enteric-retarded release synergistic design of dual-release micro-pills can adapt to the dynamic environment of the gastrointestinal tract, and the highly efficient preparation of cyclone fluidized bed microspheres reflects the improvement of the formulation quality by engineering technology innovation. However, most of the release data are based on *in vitro* dissolution tests, and there is a lack of simulation and validation of complex *in vivo* environments (e.g., intestinal pH gradient, bacterial metabolism). Furthermore, the molecular mechanisms of microcapsules/backbone materials regulating drug release (e.g., quantitative relationship between polymer dissolution kinetics and drug diffusion) have not been deeply elucidated. And most importantly, the safety of long-term administration of extended-release formulations (e.g., the effect of repeated intake of HPMC on the intestinal mucosa) and patient compliance studies are still blank.

#### 3.1.2 Controlled release preparations

The innovation of oral controlled-release technology and the integration of multifunctional nano-delivery system have significantly enhanced the targeting and therapeutic synergy of SIN for anti-RA. The enteric formulation achieves gastric protection and intestinal targeted release through coating technology, which not only reduces gastric irritation, but also regulates RA pathological processes (e.g., reducing joint damage) through intestinal flora-dependent mechanisms (Jiang Z. M. et al., 2023). Wang’s team developed an injectable nanoplatform of CIPG/SH, which breaks through the traditional delivery limitations through a multistage structural design. In detail, β-glucan microcapsules (GMs) was modified with polydopamine nanoparticles (PDAs) to enhance macrophage targeting; secondly, CIPGs were designed to co-load of indocyanine green (photothermite), catalase (ROS scavenger) and SIN to form “carrier-in-carrier” structures; thirdly, CIPGs are embedded in a thermosensitive hydrogel, and near-infrared light triggers spatiotemporally controlled drug release to synchronize inhibition of macrophage proliferation, scavenging of reactive oxygen species, and antimicrobial activity (Gong et al., 2024). The system demonstrated efficient photothermal conversion ability (>50°C warming), long-lasting thermal stability and programmed drug release properties *in vitro*, providing a new paradigm for local combination therapy of RA.

The design of the CIPG/SH system marks a leap from single controlled release to multimodal synergistic therapy for SIN delivery, even with its use of intra-articular injectable administration. However, the preparation of multi-component nano-systems is complicated (e.g., the balance between *in-situ* polymerization of PDA and retention of enzyme activity), and the feasibility of large-scale production is doubtful; meanwhile, the ability of light penetration depth to cover large human joints (e.g., knee) has not been confirmed and long-term safety data in chronic RA models are lacking; although the gut flora dependence of crocetin is mentioned (Jiang Z. M. et al., 2023), whether CIPG/SH affects the gut-joint axis has not been studied.

Oral administrations have a high degree of trust in people’s minds, but there is a need to continue to explore oral formulations of SIN that are more targeted, have fewer adverse effects, and do not harm other human organs, such as those with molecular nanocarriers (Huo et al., 2023).

### 3.2 Transdermal drug delivery

Transdermal DDS is another method of drug delivery other than oral administration or intravenous injection, and a series of new transdermal DDSs, such as liposomes, liquid crystal gels, delivery bodies, microneedles and so on, have been developed with the help of a variety of physical and chemical technologies, which are used to improve transdermal permeability and increase the bioavailability of the drug.

#### 3.2.1 Patch and ointment

Both transdermal patches and ointment can be directly applied to the skin and act on the patient site. Yang et al. combined acupoint patch therapy with patches, and by analyzing the pharmacokinetics/pharmacodynamics of SIN knee joint cavity injection, they found that acupoint injection led to a longer release of SIN and its release efficiency was higher than that of other routes of administration, which greatly improved therapeutic efficacy (Bai et al., 2022). The transdermal patch of SIN can continuously control the release rate of SIN *in vivo*, while the addition of 3% volatile oil of clove can significantly increase the transdermal penetration of SIN transdermal patch, which has a better osmotic-promoting effect (Dumitriu et al., 2023; Fan et al., 2023). However, it should not be ignored that the use of drugs, adhesives or excipients in patches and ointment can cause side effects such as rashes, local irritation, erythema or contact dermatitis, and meanwhile, it is difficult for the drug components to pass through the stratum corneum layer, which leads to a slow onset of the effect, and it usually takes a few hours after the administration of the drug to take effect.

Nanocarrier technology has demonstrated breakthrough advantages in SIN-targeted therapy for RA, overcoming the limitations of traditional therapies such as poor adherence, insufficient disease control, and systemic toxicity through precise delivery and functional synergy (Wang et al., 2021). Studies have shown that drug coupling strategies based on synovial-targeting peptides (e.g., cyclic peptide-cycloheximide complexes developed by Zhang’s team) can significantly enhance the accumulation efficiency of drugs in inflamed joints: its stable conjugation of cycloheximide A, C4-OH, with synovial-targeting peptides via 6-aminohexanoic acid linker has demonstrated excellent stability (serum/joint homogenates) and targeting selectivity *in vivo* and *in vitro*, and has been shown to be effective in alleviating acute inflammation (Zhang T. et al., 2022). Lin et al. designed graphene oxide quantum dot composite nano-systems (HA@RFM@GP@SIN NPs) to further integrate multiple functions-hyaluronic acid hybridized membranes (RFMs) to endow synovial membranes with targeting, graphene oxide quantum dots (GOQDs) to enhance the drug-carrying capacity, and to achieve macrophage dual anti-RA mechanism of M1/M2 polarization modulation and FLSs proliferation inhibition (Lin et al., 2024). The above cases corroborate the core value of nanocarriers: improving the bioavailability of insoluble drugs, prolonging the circulating half-life, reducing off-target clearance, and precisely intervening at the lesion site through controlled release of drugs (Rani et al., 2023; Logesh et al., 2023).

#### 3.2.2 Liposomes

Liposomes and their derivatives, as multifunctional nano-delivery systems, have demonstrated significant carrier advantages and technological innovations in SIN for the treatment of RA. Studies have shown that liposomes can efficiently encapsulate hydrophobic/hydrophilic drugs, such as SIN, through their bilayer structure, and enhance therapeutic efficacy and reduce systemic toxicity with the help of targeted delivery and sustained-release properties (Zakharova et al., 2023). Shen et al. developed thermosensitive liposomes (SIN-TSL) loaded with SIN-HCl to achieve a high encapsulation rate by the pH-gradient method, and combined with the microwave hyperthermia technology to achieve precise controlled release of the RA lesion and hyperthermia synergistic treatment, which significantly enhanced the anti-inflammatory effect even with the administration through tail vein (Shen et al., 2020). Wang’s team further optimized the liposome design and found that liposomes with arginine-decanoic acid ([Arg][Dec]) as vesicles had a higher encapsulation rate (83.5%), stability (90.4%), and transdermal absorption capacity (cumulative release of up to 1665.59 μg/cm^2^), and its long-lasting sustained release time was extended to 17 h, which provided an efficient vehicle for transdermal delivery of SIN (Wang et al., 2024). In addition, a novel delivery system constructed by hybridization of milk exosomes and liposomes significantly improved foot swelling, synovial lesions and inflammatory factors (TNF-α, IL-1β, IL-6) levels in CIA model rats, while solving the problem of short half-life of SIN, with an encapsulation rate of 48.21% (Wen et al., 2024). To address the need for deep delivery, the ethosome enhances stratum corneum penetration through high ethanol concentration, while the mixed monoterpene edge-activated PEGylated transfersomes utilize the dermato-compatibility of phosphatidylcholine to target SIN to the joint cavity, with intra-articular steady-state concentration and AUC_0→t_ up to 2.1-fold and 2.5-fold that of the traditional liposomes, respectively, highlighting its penetration and accumulation (Zheng et al., 2020; Akram et al., 2021; Munir et al., 2024). Together, these studies suggest that the diverse design of liposome derivatives (e.g., thermosensitive, exosome hybridization, and transfersomes optimization) can significantly enhance the delivery efficiency and therapeutic precision of SIN.

Existing studies have effectively overcome bottlenecks such as limited transdermal absorption, short half-life and insufficient joint targeting of SIN through strategies such as thermal response, fatty acid optimization, exosome hybridization and transfersomes penetration enhancement. Among them, the synergistic application of thermosensitive liposomes and microwave thermotherapy, the efficient delivery of MMPTs to the joint cavity, and the immunomodulatory effect of exosome hybridization system exemplify the innovative value of interdisciplinary technology integration. However, the clinical translational potential of most liposomal systems is limited by the complexity and cost control of the scale-up production process; in addition, the long-term biosafety (e.g., immunogenicity) of exosome-liposome hybrid systems has not yet been adequately validated; and, second, the comparison of the delivery efficiencies of different liposomal derivatives (e.g., ethosomes vs. transfersomes) in the microenvironment of RA remains to be investigated.

#### 3.2.3 Hydrogels

**FIGURE 2:**
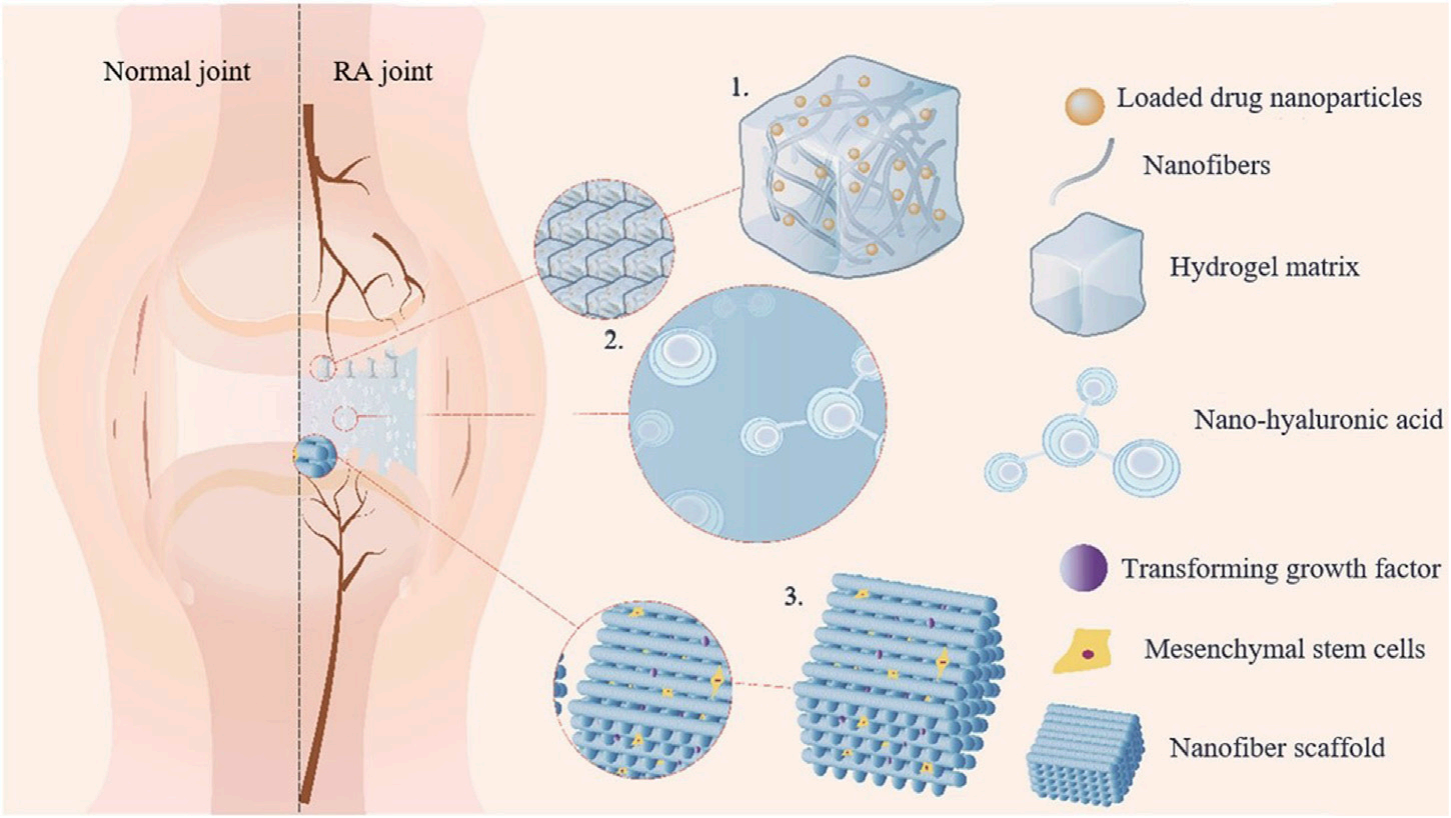
Schematic diagram illustrating the mechanism of nanocomposite hydrogel in repairing joint inflammation. **(1)** The application of drugs to target cells for synergistic effects; **(2)** The simulation of joint environment to reduce friction; **(3)** The loading of seed cells onto the nanofiber scaffold to induce chondrocyte differentiation.

Gel delivery systems have emerged as a novel carrier strategy for SIN in the treatment of RA by virtue of its three-dimensional network structure, excellent biocompatibility and slow drug release properties. Studies have shown that nanocomposite hydrogel-based delivery systems can promote cartilage repair by modulating the inflammatory microenvironment of joints (Figure 2), providing a potential solution for osteoarticular protection in RA (Tian et al., 2024). To address the bottleneck of transdermal absorption of SIN, the lipid-based liquid crystal gel significantly enhanced the skin permeability of SIN by introducing the lipophilic pro-osmotic agent cinnamaldehyde, and its two-component cubic liquid crystal structure simultaneously realized the efficient co-loading of hydrophilic/lipophilic drugs, and the drug release conformed to the Fick diffusion-dominant Higuchi equation, which revealed the universality of this system for transdermal co-administration of SIN (Chen J. et al., 2022). The SIN microemulsion gel (SMG) developed by Ding’s team verified its slow-release advantage by two-site microdialysis: after transdermal administration, the local drug concentration in the skin (C_max_: 10.91 ± 3.05 μg/mL) was significantly higher than that in the blood (C_max_: 6.74 ± 1.91 μg/mL), and the retention time of the skin drug (T_max_: 180 min) was higher than that in the systemic exposure (T_max_: 240 min), suggesting that SMG can form a long-lasting reservoir effect in the skin (AUC skin/blood ratio of 1.73:1), thus extending the local therapeutic (Ding et al., 2022). Tian et al. further optimized the liposomal gel technology and confirmed that the SIN liposomal gel had superior slow-release performance and antioxidant activity compared with the traditional oral drug delivery, and its liposome-hydrogel composite system effectively enhanced the drug stability and the target tissue accumulation capacity through the synergistic effect of dual carriers (Tian, 2021; Yang et al., 2022). Together, these studies indicate that the rational design of composite carriers (e.g., liquid crystal phase, microemulsion, and liposome) can break through the limitations of a single gel carrier, and significantly enhance the efficiency and therapeutic targeting of transdermal delivery of SIN.

The current innovative research on gel delivery systems has successfully solved the problems of low transdermal absorption and rapid metabolism of traditional dosage forms of SIN, especially the osmotic-promoting design of liquid crystal gel, the reservoir effect of microemulsion gel and the antioxidant synergistic mechanism of liposomal gel, which demonstrates the potential of multi-dimensional delivery optimization. The advanced research methodology (e.g., two-site microdialysis technology) provides precise data support for the drug release kinetics *in vitro* and *in vivo*, while the study of the correlation between the nanocomposite hydrogel and the repair of the bone and joint microenvironment expands the scenarios for the application of SIN in RA bone destruction intervention. However, the long-term biocompatibility of the composite carrier (e.g., the effect of liposome residues on synovial tissues) and the synergistic mechanism of gel co-delivery of SIN with other anti-RA drugs (e.g., methotrexate) need to be further investigated.

#### 3.2.4 Microneedles

Microneedles combine the features of conventional injections and patches, and are a new technology for transdermal drug delivery that can significantly improve drug availability, and the types can be divided into solid-type, drug-coated, and drug-carrying dissolution microneedles (Hou et al., 2023; Hua et al., 2024). Studies have shown that the microneedle systems based on SIN are diverse in design and remarkable in functionalization. Wang et al. developed a near-infrared responsive SIN-HCl reservoir microneedle, which could achieve a drug loading capacity of 0.5 mg/cm^-2^ and precisely deliver SIN to dermis layer of the skin with a puncture depth of 300 μm to avoid damaging the deeper tissues, with a cumulative release rate of 74.3% at 24 h, which is in line with a first-degree kinetic (Wang et al., 2022). In addition, ROS-responsive nanoparticles coupled with fucoidan microneedle system (FTL@SIN MNs) synchronously alleviated synovial inflammation and promoted cartilage repair by modulating macrophage polarization (M1 to M2 conversion) and pro-inflammatory factor secretion, while the fucoidan carrier enhanced the mechanical strength and stability of the FTL@SIN MNs (Liu X. et al., 2024). Chen’s team further combined ROS-responsive nanotechnology to construct a microencapsulated dissolvable microneedle (B/S-TM@MN) co-loaded with berberine and SIN, and achieved synergistic release of the two drugs through PLGA-TK-PEG self-assembly, which significantly inhibited synovitis (reduction of CD68^+^ macrophages) and neovascularization (reduction of CD31+vessel density) in CIA mice with superior efficacy to monotherapy (Hua et al., 2025). The two-layer polyvinylpyrrolidone (PVP)/*Phaseolus lunatus L.* polysaccharide (PLP) microneedles (SIN@PLP MNs) designed by Zhang et al. realized the biphasic release of SIN: the PVP layer was rapidly released (within 5 min) to exert immediate analgesic effect, and the PLP layer was continuously released (12 h) to maintain the anti-inflammatory effect by inhibiting the NF-κB/MAPK pathway and FLS proliferation, which effectively alleviated cartilage erosion and inflammatory pain in the AIA model (Feng et al., 2025). The above studies collectively demonstrated that the functionalized microneedle system significantly enhanced the anti-RA efficacy and patient compliance of SIN through spatiotemporally controlled drug release, synergistic multi-targeted interventions, and delivery vehicle optimization.

The integration of microneedle technology with SIN marks the leap from single drug intervention to intelligent and precise delivery mode for RA treatment. Through the innovation of material engineering and nanotechnology, the existing studies have successfully solved the problems of low bioavailability and short half-life of SIN in the traditional delivery mode, and at the same time endowed them with the characteristics of ROS-responsive, dual-drug synergism, and biphasic release, which have significantly enhanced the concentration of the local drug in the joints and the durability of the action. In particular, the study highlights the multi-modal advantages of microneedle system in regulating the RA pathological microenvironment: on the one hand, it can achieve multi-dimensional anti-inflammation by targeting macrophage polarization, FLS proliferation inhibition, and inflammatory pathway blockage; on the other hand, it can enhance the mechanical properties and biocompatibility by using fucoidan and PLP, which lays the foundation for clinical translation. However, the current study still faces the following challenges: first, the safety of microneedles in long-term skin retention and the effects of repeated administration on the skin barrier have not been systematically evaluated; second, most of the ROS-responsive designs rely on the validation of *in vitro* models, and the efficiency of their response in complex *in vivo* microenvironments (e.g., synovial hypoxia, matrix metalloproteinase enrichment) needs to be further investigated; third, the optimization of the dosage ratio for dual- or multidrug combinations and the pharmacokinetic synergistic mechanism still need to be thoroughly investigated. Third, the optimization of the dose ratio and pharmacokinetic synergistic mechanism of dual- or multi-drug combinations still need to be deeply analyzed.

#### 3.2.5 Electroporation

Electroporation is a microbiology technique in which an electric field is applied to a cell to increase the permeability of the cell membrane, thereby allowing chemicals, drugs, or DNA to be introduced into the cell, and is commonly used in microbiology research (Kougkolos et al., 2024). Skin electroporation for drug delivery utilizes electrical impulses to loosen skin structures, increase skin cell gaps and epidermal fissures, and disrupt skin barrier function in a temporary and non-invasive manner to increase drug absorption.

Although the targeted transdermal DDS shows theoretical advantages in the SIN delivery, its clinical efficacy is still limited by factors such as biological barriers and immature technologies. Collaborative innovation in bio-responsive materials (e.g., pH/ROS/MMP responsive materials precisely release drugs), physical enhancement technologies (e.g., microneedles/electroporation break through the physical barrier), and precise manufacturing (e.g., exosomes mediate ultimate synovial membrane targeting) is crucial for developing intelligent responsive trans-barrier DDSs to achieve truly targeted treatment for synovial membranes. Therefore, the future research direction should focus on co-development of diagnostics and DDSs, ultimately enabling patient-tailored RA treatment.

### 3.3 Intra-articular injection administration

Compared with oral administration, intra-articular injection (IAI) administration can deliver the drug directly to the joint cavity, reduce the administered dose, avoid systemic exposure and potential adverse effects (Ma et al., 2022), and enhance the precision and long-lasting efficacy of SIN in the treatment of RA. Studies have shown that compared with traditional oral or IAI therapies (e.g., diclofenac sodium, methotrexate), SIN can target and regulate synovial inflammation and reduce systemic toxicity via IAI, but its efficacy is limited by the rapid clearance of small molecules in the joint cavity (Wang et al., 2021; Huang et al., 2022). To address this bottleneck, multiple functionalized delivery systems have been developed: Yang et al. enhanced the bioavailability of SIN to 2.0-fold of the injectable solution by liposome-thermosensitive gel conjugation technology, prolonged the time to peak by 3.0-fold, and reduced the peak blood concentration (P < 0.05), which resulted in long-lasting and slow-release with pharmacokinetic optimization (Wang et al., 2020). In addition, nanocrystalline self-stabilized Pickering emulsion with solid liposome nanoparticles for IAI further reduced toxicity and improved bioavailability by forming a local drug reservoir (Zhang J. et al., 2022; Zhang et al., 2023). Furthermore, Niu et al. compounded a SIN microemulsion (SIN-ME) with poloxamer thermosensitive gel to construct an injectable sustained-release system (*T*_sol-gel_: 35.2°C ± 1.33°C), which achieved a cumulative drug release rate of 66.7% ± 6.2% at 48 h, significantly prolonging the intra-articular retention time (Niu et al., 2024). The ROS-sensitive hyaluronic acid nanocarriers (PAM-HA@SIN NPs) developed by Shang et al. by virtue of the bone adsorption and ROS scavenging function, they extended the retention time of SIN in inflamed joints to more than 20 days and significantly outperformed commercialized available free SIN through a combined antioxidant/anti-inflammatory effect (Shang et al., 2023). The team of Wang designed a bionic-DDS (CIPG/SH) breaks through the limitations of traditional single drug release modes by integrating photothermal/photodynamic properties with thermal controlled release characteristics to achieve synergistic macrophage proliferation inhibition and antimicrobial therapy triggered by the near infrared (Gong et al., 2024). The above system has systematically solved the key technical problems of IA delivery of SIN through material engineering and drug release mechanism innovation.

The functionalized design of the intra-articular injection delivery system provides a breakthrough strategy for the RA-targeted therapy of SIN. Existing studies have successfully overcome the core challenges of rapid intra-articular drug clearance and insufficient local concentration through thermosensitive gel slow release, bionic carrier integration and nanotechnology optimization. Among them, the long-lasting retention properties of PAM-HA@SIN NPs and the photothermal/photodynamic synergistic effect of CIPG/SH exemplify the innovativeness of multimodal therapeutics, while the optimization of the slow-release kinetics of the liposome-thermosensitive gel provides an important reference for the clinical efficacy/safety balance. However, the *in vivo* validation of most current delivery systems is limited to rodent models, whose joint cavity volume and synovial fluid composition differences from humans may affect the clinical translational effect; second, the long-term intra-articular biocompatibility of thermosensitive gels or nanocarriers (e.g., effects on cartilage metabolism) has not been fully evaluated.

## 4 Summary and discussion

RA is a chronic autoimmune disease characterized by synovitis, cartilage and bone damage, and high disability rate. Currently, no specific drug treats RA in clinic, management focuses on prevention and control. As a transforming DMARD, SIN has been used to treat rheumatoid arthritis over 25 years in China. SIN targets RA pathogenesis at multiple levels: (1) Regulating immune cells, reducing macrophage M1 polarization, dendritic cell activation, and T-cell proliferation; (2) Inhibiting inflammatory cytokines by blocking NF-κB, MAPK, and NLRP3 pathways, decreasing pro-inflammatory and increasing anti-inflammatory mediators; (3) Suppressing synovial hyperplasia and angiogenesis by inhibiting FLS proliferation and migration, and VEGF-driven neovascularization; (4) Mitigating bone erosion by reducing osteoclastogenesis and correcting TRAP, MMP9, and RANKL/OPG imbalances, preserving bone integrity in preclinical models.

Innovative DDSs have improved SIN’s clinical use. Oral formulations like sustained-release tablets enhance bioavailability and reduce dosing frequency, despite limitations from hepatic first-pass metabolism. Transdermal systems, including microneedles, lipid-based carriers, and hydrogels, allow localized drug accumulation, extended release, and reduced systemic toxicity. Intra-articular injectables provide sustained synovial drug retention by targeting the inflammatory environment of RA. These advancements demonstrate the synergy between SIN’s pharmacology and advanced DDSs in addressing RA’s complex pathophysiology.

In addition, while SIN effectively treats RA, histamine-releasing anaphylactoid reactions (HRARs) often occur in some patients, presenting as significant hypothermia, increased skin vascular permeability, lung tissue damage, and increased infiltration of mast cells and IL-33 expression in skin and lung tissues (Huang et al., 2017). It is great significance to establish effective clinical protocols to manage such HRARs. Therefore, various pharmaceutical strategies have been explored to address these deficiencies, such as prolonged release behaviors, enhanced skin penetration and adsorption for transdermal delivery, targeted SIN delivery using new materials or conjugates, and co-amorphous technology (Chen et al., 2022b). For instance, Chen et al. co-amorphized SIN with the antihistamine terlisperone (TRA) and successfully obtained a series of co-amorphous samples. The results showed that these samples achieved sustained SIN release without adverse reactions caused by histamine-release (Chen et al., 2022c). Therefore, mast cell membrane stabilizers and H1 receptor blockers are recommended for effective prevention SIN-induced HRARs, such as sodium cromoglicate, cetirizine, and TRA, which can be used in combination with SIN for clinical treatment of HRARs caused by SIN.

Numerous clinical trials have demonstrated that the combination of SIN with DMARDs (primarily MTX and LEF) enhances both clinical efficacy and safety of SIN in treating RA patients. In Huang’s study, patients were randomly assigned at a 3:2 ratio to receive MTX combined with SIN, and the efficacy and safety were assessed at weeks 4, 12 and 24. The results: showed that in the intention-to-treat (ITT) analysis, 65.3% of patients treated with MTX+SIN showed improved disease activity as determined by the ACR50 response at week 24 compared to 69.6% of patients treated with MTX+LEF. And significant reductions (p < 0.05) in gastrointestinal adverse reactions and liver toxicity were found in patients treated with MTX+SIN, so MTX+SIN combination therapy is probably one of the choices for treating RA (Huang et al., 2019). What’s more, it can be seen from Kour’s review article that the MTX combination with SIN for RA therapy had shown promising results both in experimental arthritic models and clinical arthritis, and might be superior in terms of controlling adverse drug reactions (Kour et al., 2021).

## 5 Challenges in translating SIN and SIN-based DDSs to clinical use

The clinical translation of SIN, a natural candidate for the treatment of RA, faces multiple and complex challenges. At the regulatory level, compositional complexity (e.g., alkaloid diversity and unknown impurities) requires strict adherence to the ICH Q3D guidelines for elemental impurities, and batch consistency (RSD<2%) is ensured by HPLC-MS fingerprinting. Currently, there are significant differences in global regulatory pathways: Europe and the United States require the completion of phase I-III clinical trials based on botanical drugs (e.g., FDA botanical drug pathway), while China can register new drugs based on TCM, but with sufficient structural modifications. The intellectual property rights are still unfriendly for natural compound patents, but in favor of patents on formulation compound patents (e.g., HA@M@PB@SIN NPs) and medical use patents.

The non-negligible risk of toxicity is another major obstacle to the clinical translational use of SIN-based preparations. Dose-dependent hepatotoxicity and histamine release-induced adverse effects are particularly prominent, which are associated with the inhibition of CYP3A4 by the metabolite nortriptyline quinine leading to self-accumulation (Li et al., 2023). Meanwhile, preclinical trials of long-term biocompatibility (the current clinical trials have a maximum duration of only 24 weeks, lacking assessment of bone metabolism effects for a period of ≥ 52 weeks and a two-year carcinogenicity study) and assessment of dynamic changes in the RA inflammatory microenvironment (e.g., pH, reactive oxygen species, protease levels) remain deficient. Therefore, the development of smart DDSs integrating multi-stimulus responsiveness and real-time detection with hierarchical toxicity control strategies (e.g., CYP450 antagonism with glycyrrhizic acid, pre-administration of H1 receptor antagonists, etc.) is of great clinical significance. Furthermore, use of exosomes, cell membranes, cells, and extracellular matrices to construct nano-biomimetic DDSs (Li et al., 2022; Qu et al., 2023; Gan et al., 2024), which is expected to minimize the toxicity of nanomedicines and enhance the safety and efficacy of formulations. Nevertheless, the challenges related to material sourcing for bio-nanocarriers, along with the associated ethical controversies, represent significant research bottlenecks that must be addressed to enable large-scale application.

The combination therapy utilizing SIN, a type of phytochemical, alongside DMARDs has demonstrated synergistic therapeutic effects while mitigating the toxic side effects associated with current RA treatments. This includes improvements in solubility and permeability, as well as addressing challenges related to pH-dependent degradation, first-pass metabolism, P-glycoprotein-mediated efflux, and low oral bioavailability (Kour et al., 2021). Nevertheless, a critical consideration in this combination therapy is the potential pharmacokinetic and pharmacodynamic interactions between DMARDs and SIN. The concurrent use of these agents may lead to significant alterations in drug concentrations, potentially elevating the plasma levels of DMARDs and thereby exacerbating toxicity. Consequently, the design and rational selection of agents for combination drug delivery in RA should prioritize the identification of safety concerns related to enhanced toxicities and pharmacokinetic interactions.

In summary, ongoing advancements in drug formulation and pharmaceutical excipients, along with scientists’ persistent efforts, are expected to lead to the creation of safer and more effective SIN dosage forms. These innovations are anticipated to soon enable industrial production and widespread use in RA treatment, thereby enhancing the role of TCM in managing inflammatory diseases.
