# Correction: Research progress in treatment of rheumatoid arthritis with Sinomenine and related formulations based on different administration routes

**DOI:** 10.3389/fphar.2025.1685111

**Published:** 2025-09-09

**Authors:** Wenya Wang, Zihui Wang, Aixia Ling, Chunyan Zhang, Mei Lv, Lufen Huang, Yanlian Niu

**Affiliations:** School of Pharmacy, Jining Medical University, Rizhao, China

**Keywords:** Sinomenine, mechanism of action, related formulations, drug delivery system, rheumatoid arthritis

There was a mistake in the caption of **Figure 2** as published. Part labels “A,C,E” and “B,D,F” were given, when the labels are “1”, “2”, and “3” according to the figure graphic. The following sentence was also erroneously included (“Reproduced with permission from Wu et al., AAPS PharmSciTech; published by Springer Nature Link, 2024”), and as such has been removed. The corrected caption of **Figure 2** appears below.

“Schematic diagram illustrating the mechanism of nanocomposite hydrogel in repairing joint inflammation. (**1**) The application of drugs to target cells for synergistic effects; (**2**) The simulation of joint environment to reduce friction; (**3**) The loading of seed cells onto the nanofiber scaffold to induce chondrocyte differentiation.”

The original article has been updated.

